# The ubiquitin-like modifier FAT10 is not essential for MHC-I antigen presentation

**DOI:** 10.3389/fimmu.2025.1636951

**Published:** 2025-08-01

**Authors:** Natalie Pach, Sarah Ochs, Jinjing Cao, Julia Ottlinger, Annette Aichem, Michael Basler

**Affiliations:** ^1^ Institute of Cell Biology and Immunology Thurgau (BITG) at the University of Konstanz, Kreuzlingen, Switzerland; ^2^ Division of Immunology, Department of Biology, University of Konstanz, Konstanz, Germany

**Keywords:** FAT10, UBD, proteasome, MHC-I, antigen processing, antigen presentation, cytotoxic T cells

## Abstract

**Introduction:**

The presentation of pathogen-derived antigens on major histocompatibility complex (MHC) class I is crucial for the antiviral immune response. Degradation of intracellular pathogen-derived proteins by the 26S proteasome generates peptides that can be loaded on MHC-I molecules and presented to cytotoxic T cells. The cytokine-inducible ubiquitin-like modifier (ULM) HLA-F adjacent transcript 10 (FAT10) is encoded in the MHC locus and targets its substrates for proteasomal degradation. Therefore, it acts as an alternative signal for protein degradation, indicating a role in generating the peptide pool for MHC-I presentation. In this study, we aimed to elucidate the role of FAT10 in MHC class I presentation.

**Methods:**

Using different human and mouse cell lines deficient for FAT10, the effect of FAT10 on MHC-I surface expression and recovery was studied. For the evaluation of antigen presentation of viral and endogenous epitopes, T cell hybridoma assays and flow cytometry analysis were used.

**Results:**

In our study, using model antigens and FAT10-deficient cells, we found that the absence of FAT10 does not affect the abundance of MHC-I molecules or the generation of endogenous and virus-derived MHC-I epitopes. Furthermore, we demonstrated that the cytotoxic T cell response to different viruses remains unchanged in FAT10-deficient mice compared to wild-type mice.

**Discussion:**

In summary, our findings indicate that the lack of FAT10 does not impact antigen presentation or the cytotoxic T-cell response across a number of different MHC-I-restricted peptides. Hence, we conclude that the contribution of FAT10 to MHC-I antigen presentation has previously been overestimated.

## Introduction

Intracellular peptides are presented on MHC class I molecules resulting in the activation of cytotoxic lymphocytes (CD8^+^ T cells). The generation of peptides presented on MHC class I molecules strongly depends on the ubiquitin-proteasome system (1). Lysine (K)-48 linked polyubiquitin chains mark a substrate for degradation by the 26S proteasome. This ubiquitin conjugation requires a series of specific enzymes: E1 (activating enzyme), E2 (conjugating enzyme), and E3 (ligase), which are the key players in transferring ubiquitin chains to target proteins.

Ubiquitin-like modifiers (ULMs) exhibit structural similarities to ubiquitin and can regulate diverse biological processes. The HLA-F-adjacent transcript 10 (FAT10) is a ULM targeting proteins for degradation through a mechanism that is independent of ubiquitin (2–4). The modifier is predominantly expressed in cells of the immune system but can also be upregulated in various cell types in response to the pro-inflammatory cytokines TNF and IFNγ (5–7). Furthermore, its expression is increased during the later phase of dendritic cell (DC) maturation (8, 9). Similar to ubiquitin, FAT10 is covalently conjugated via an isopeptide bond to lysine residues within a substrate. This process, referred to as FAT10ylation, is facilitated by an enzyme cascade involving the bispecific E1 activating enzyme UBA6 and the E2 conjugating enzyme USE1 (UBA6-specific E2 enzyme) (10–13). Interestingly, under inflammatory conditions in the presence of TNF, FAT10 conjugation appears to be independent of USE1 (14). In contrast to ubiquitin, FAT10 has a notably short half-life of approximately 1 h and is subjected to degradation along with its substrate (3, 15). This differs from the fate of ubiquitin, which is removed by deubiquitinases (DUBs) prior to the substrate entering the proteasome (3, 15, 16).

FAT10 was shown to be involved in several biological processes including NF-κB activation, apoptosis and cell proliferation (17). FAT10-deficient mice are viable and show minimal phenotypical differences compared to wild type mice (18). However, FAT10-deficient mice have an extended lifespan and reduced adiposity, indicating a role of FAT10 in metabolic regulation (19). At cellular level, lymphocytes from FAT10^-/-^ mice demonstrate increased susceptibility to apoptosis (18). Given that FAT10 is located within the MHC class I region and is involved in protein degradation pathways, it may play a critical role in MHC-I antigen presentation. The degradation rate of FAT10ylated substrates can be significantly increased by the ubiquitin-like protein NEDD8 ultimate buster-1 long (NUB1L) (20). Here, the N-terminal domain of FAT10 interacts with the ubiquitin-associated (UBA) domain of Nub1. Nub1 binds through its UBA domain the VWA domain of the 19S cap subunits Rpn10, or to Rpn1 bringing FAT10ylated proteins in close proximity to the proteasome (21).

*In vitro* experiments have demonstrated that N-terminal modification of viral proteins with FAT10 leads to an enhanced MHC-I presentation of peptides derived from these viral proteins (22–25). Furthermore, the peptidome of FAT10 overexpressing cells differed from FAT10-deficient cells. These data indicate a functional role of FAT10 in contributing to the peptide pool available for MHC class I presentation. However, to which extent FAT10 is involved in generation of peptides presented on MHC-I under endogenous conditions remains to be elucidated. In this study, we aimed to investigate the role of FAT10 in antigen presentation on MHC class I molecules both *in vitro* and *in vivo*. Using distinct immunogenic peptides, FAT10-deficient mouse and human cells, as well as FAT10^-/-^ mice, we studied whether FAT10 influences alternative proteasomal targeting for MHC-I restricted antigen presentation.

## Results

## Absence of FAT10, UBA6 or USE1 does not affect MHC-I surface expression

**Figure 1:**
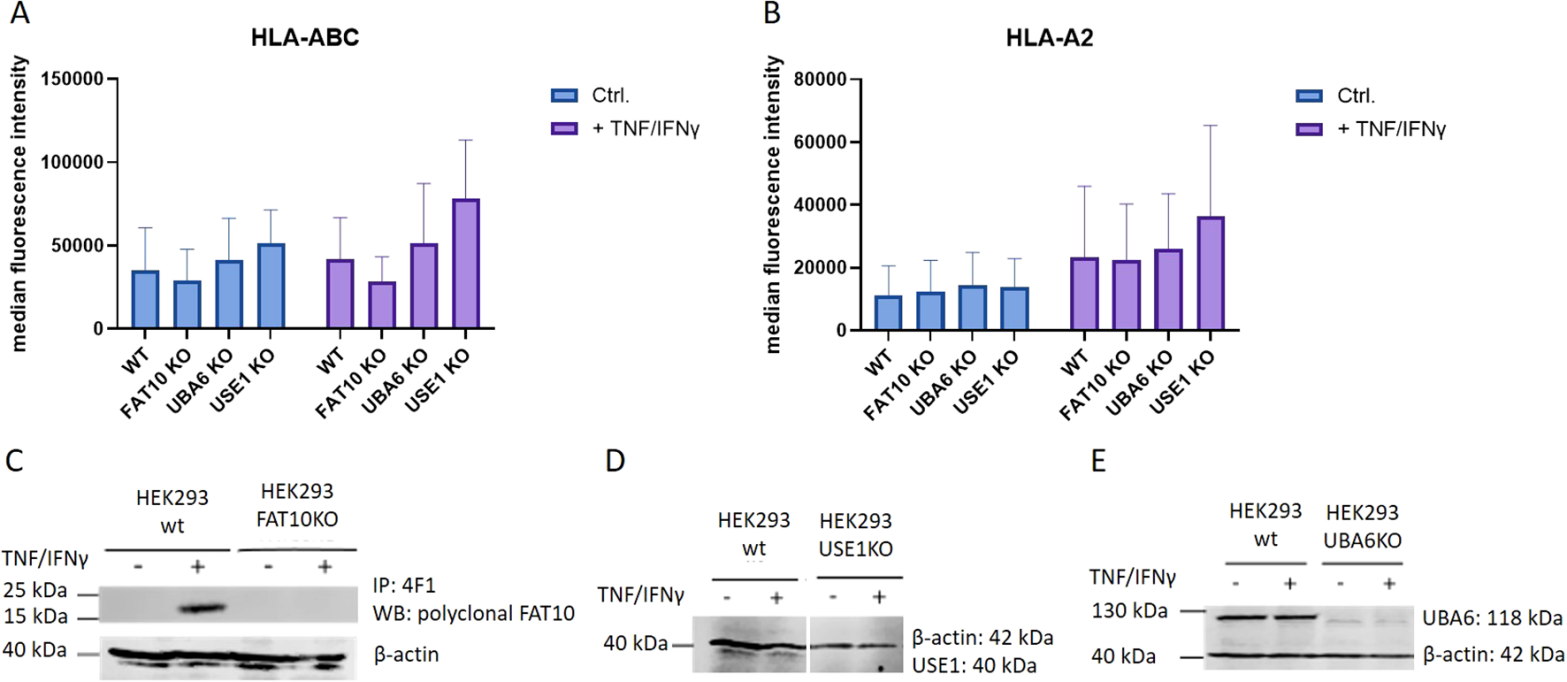
MHC class I surface expression in HEK293 cells. HEK293 cells and different KO clones with deleted genes for FAT10, UBA6, or USE1 were tested for MHC class I surface expression. Where indicated (+), cells were treated with TNF/IFNy for 24h. **(A, B)** MHC-I surface expression was measured by flow cytometry using antibodies recognizing pan human MHC-I **(A)** or restricted to the HLA-A2 allele **(B)**. Data are shown as mean median intensity ± SD derived from 4 independent experiments measured in duplicates. **(C)** FAT10 was immunoprecipitated from wild type (wt) or FAT10-deficient (FAT10KO) HEK293 cells with the 4F1 antibody and analyzed by western blot using polyclonal antibodies recognizing FAT10. **(D, E)** Western blot analysis of USE KO cells **(D)**, or UBA6 KO cells **(E)**. β-actin was used as loading control.

Since the ubiquitin-like modifier FAT10 is encoded in the major histocompatibility complex class I locus (26), this suggests a potential role in antigen presentation. To address the question of whether FAT10 is involved in this process, the surface expression of MHC-I on HEK293 cells was investigated. MHC-I surface expression of wild type cells was compared to FAT10-deficient cells. Additionally, HEK293 cells lacking the FAT10 E1 enzyme UBA6 and the FAT10 E2 enzyme USE1 were included in the analysis. These enzymes are essential for FAT10 conjugation, and mutation of their catalytic subunit prevents FAT10 from covalently being attached to its substrates, thereby inhibiting FAT10-mediated proteasomal degradation (10, 12). Given that the basal expression level of FAT10 in HEK293 cells is typically low, we induced its expression using the pro-inflammatory cytokines TNF and IFNγ. Flow cytometry analysis showed no significant differences in MHC class I expression on the cell surface between wild type (wt) cells and different knockout (KO) cells (**Figures 1A, B**). Treatment with TNF and IFNγ induced a notable increase in MHC expression across all cell lines, however, MHC-I surface expression was not significantly altered in the absence of FAT10, UBA6, or USE1. To confirm the knockout of the targeted genes, western blot analysis was performed to assess the expression levels of FAT10, UBA6, and USE1 at the protein levels (**Figures 1C–E**). FAT10 expression in HEK293 cells analyzed by FAT10 immunoprecipitation followed by western blot analysis was only detectable upon induction with TNF and IFNγ (**Figure 1C**). Absence of FAT10, UBA6, and USE1 could be confirmed in the respective knockout clones. In contrast to FAT10, the expression levels of the enzymes responsible for the conjugation of FAT10 remained unchanged upon TNF/IFNγ treatment. In summary, the absence of FAT10 and its associated conjugation machinery did not alter the MHC-I surface expression levels in HEK293 cells.

## Absence of FAT10 in different human cell lines does not alter MHC-I surface expression

**Figure 2:**
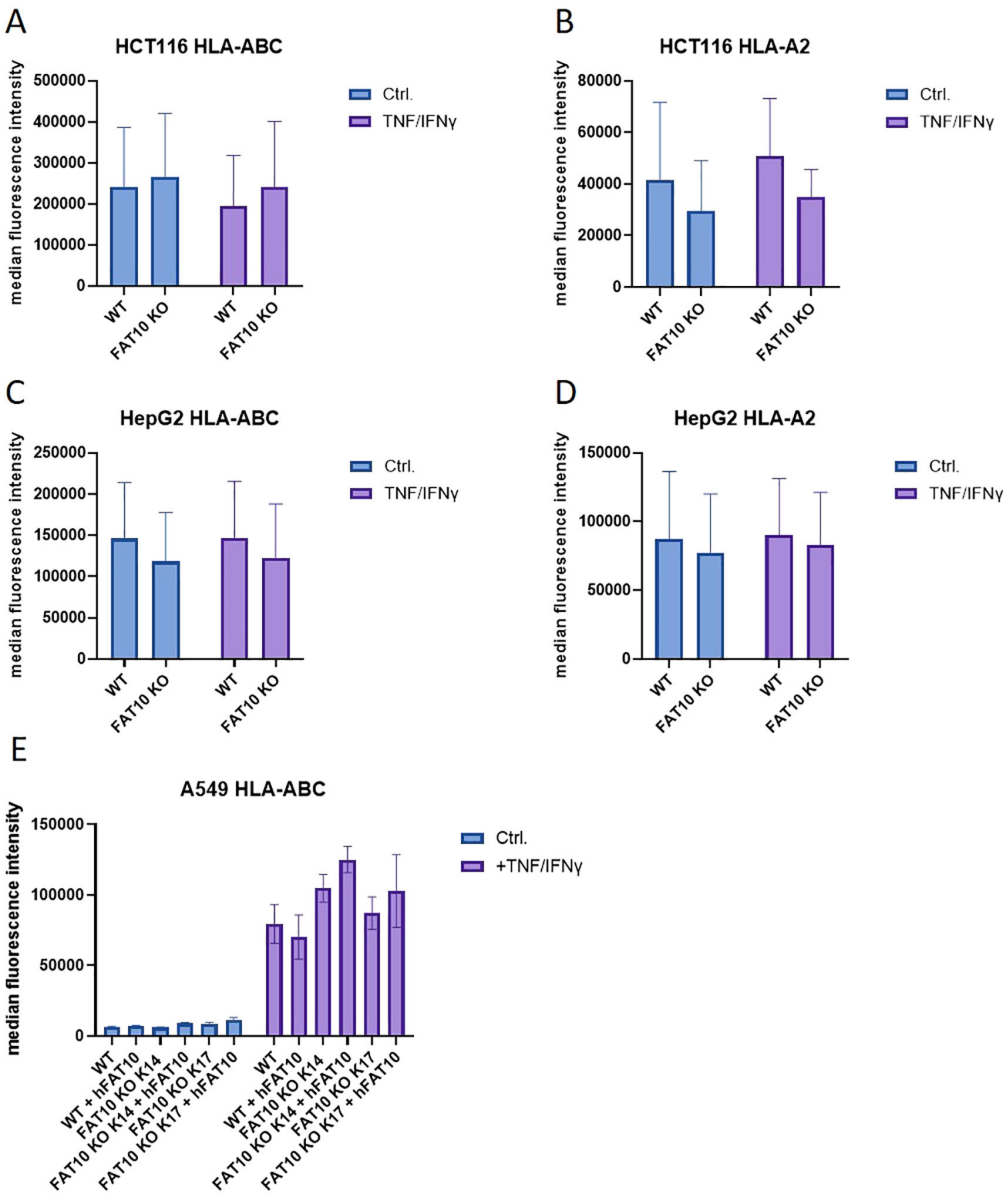
MHC class I expression in different human cell lines. **(A–D)** HCT116 **(A, B)**, HepG2 **(C, D)** and A549 **(E)** cells and their respective FAT10 KO lines were analyzed for MHC class I expression. Where indicated, cells were treated with TNF and IFNγ for 24 h. MHC surface expression was determined by flow cytometry using antibodies recognizing pan human MHC molecules **(A, C, E)** or restricted to the HLA-A2 allele **(B, D, E)**. Wild type (WT) or FAT10 deficient (clone 14 or clone 17) A549 cells were transduced with a lentivirus encoding human FAT10 (hFAT10). HLA-A, B,C surface expression was determined by flow cytometry. **(A–E)** Data are shown as mean median intensity ± SD derived from 3 independent experiments measured in duplicates.

Initial experiments were performed with HEK293 cells. To exclude cell-type specific effects, we further examined MHC-I expression in three different human cell lines: the colon carcinoma cell line HCT116, the hepatocellular carcinoma cell line HepG2, and the lung cancer cell line A549. Absence of FAT10 in knockout cells was confirmed using immunoprecipitation followed by western blot (**Supplementary Figure 1**). Without TNF/IFNγ treatment FAT10 could not be detected in all three cell lines. HCT116 and HepG2 cells were stimulated with TNF/IFNγ and HLA-A,B,C and HLA-A2 MHC class I surface expression was evaluated using flow cytometry (**Figure 2**). Consistent with HEK293 cells (**Figure 1**), the absence of FAT10 did not alter the surface expression of MHC class I molecules. Interestingly, TNF/IFNγ treatment did not enhance MHC class I expression in HCT116 and HepG2 cell lines regardless of whether pan human MHC molecules (**Figures 2A, C**) or the HLA-A2 allele (**Figures 2B, D**) were considered. On unstimulated A549 cells, surface MHC class I molecules could hardly be detected, but could be drastically increased after treatment with TNF/IFNγ (**Figure 2D**). However, also in A549 cells no difference in MHC-I surface expression could be observed between FAT10-proficient and FAT10-deficient cells. Furthermore, A549 wild type cells and knockout cells (clone 14 and 17) were transduced with a lentivirus encoding FAT10. The reintroduction of FAT10 into the different A549 cells did not significantly alter MHC class I expression. Consequently, these findings suggest that absence or overexpression of FAT10 does not influence the steady-state levels of MHC class I on the cell surface of 4 different human cell lines.

## Absence of FAT10 does not influence reappearance of surface MHC-I expression

**Figure 3:**
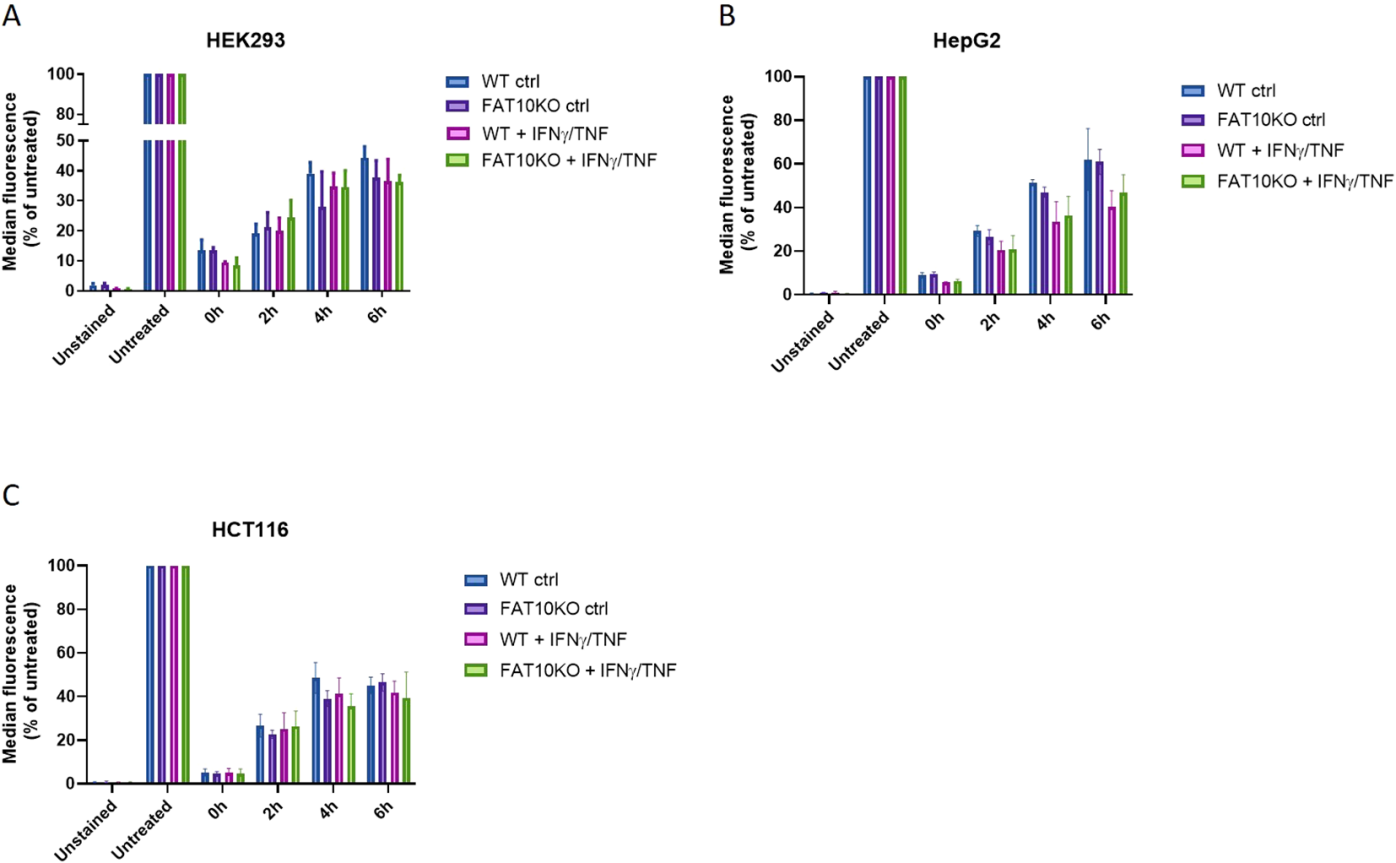
Influence of FAT10 on reappearance of MHC-I-peptide complexes after acid wash treatment. **(A–C)** HEK293 **(A)**, HepG2 **(B)**, HCT116 **(C)** and their respective FAT10 KO clones were analyzed for MHC class I recovery after acid wash treatment. Where indicated, cells were treated with TNF/IFNγ for 24 (h). Total human MHC-I surface expression (HLA-A,B,C) was determined by flow cytometry at indicated time points post acid wash treatment. Untreated cells were set to 100% of MHC-I surface expression and served as a reference for maximal MHC-I surface expression. Unstained cells were used as negative control. Shown is the mean ± SD of 3 different experiments measured in duplicates.

So far, the MHC-I surface expression in the presence and absence of FAT10 was analyzed at steady-state conditions. To further investigate the role of FAT10 in MHC-I presentation, we examined its involvement in the reconstitution of MHC class I-antigen complexes following their removal from the cell surface via an acid wash, when endogenous antigen might become limiting (**Figure 3**). HEK293, HepG2 and HCT116 cells and their corresponding FAT10 KO clones were stimulated with TNF/IFNγ to induce FAT10 expression for 24 h, or were left untreated. Cells were exposed to an acid wash treatment and the recovery of HLA-A,B,C was quantified by flow cytometry. Acid wash treatment reduced the surface MHC-I level by over 90% for all cell types examined (**Figures 3A–C**). In the period of 6 h, a recovery of MHC class I molecules could be observed, reaching up to 50% of baseline levels. Treatment with TNF/IFNγ did not significantly influence the re-appearance of MHC class I-antigen complexes on the cell surface. Importantly, the absence of FAT10 does not impact the recovery of peptide-MHC class I complexes following acid wash treatment in any of the analyzed cell lines.

## Absence of FAT10 does not alter MHC-I surface expression of different lymphocyte populations

**Figure 4:**
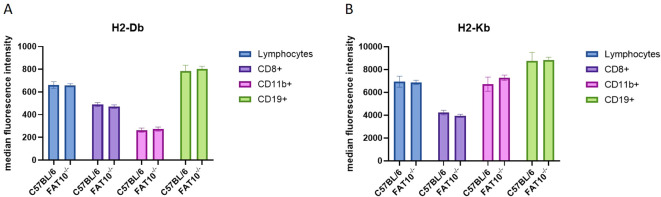
MHC class I expression on mouse immune cells. **(A, B)** Indicated subsets of immune cells derived from splenocytes of C57BL/6 or FAT10 KO mice were analyzed for H2-D^b^ **(A)** or H2-K^b^ **(B)** expression by flow cytometry. Data are shown as median fluorescence intensity ± SD derived from 5 mice measured in duplicates.

So far, the expression levels of MHC class I molecules on the cell surface was studied in different human cancer cell lines. Next, we wanted to assess the impact of FAT10 on MHC class I expression on different mouse immune cell types. Therefore, splenocytes from C57BL/6 mice or FAT10 knockout mice (FAT10^-/-^) were used to evaluate the expression of H2-D^b^ (**Figure 4A**) and H2-K^b^ (**Figure 4B**) on CD8^+^ (cytotoxic T cells), CD11b^+^ (macrophages and monocytes), and CD19^+^ (B cells) cells by flow cytometry. The absence of FAT10 did not affect the abundance of MHC-I molecules on the surface of total lymphocytes or the different immune cell populations examined. These results indicate that the absence of FAT10 does not influence steady-state MHC-I expression on different immune cell types derived from naïve mice. Notably, whereas FAT10 can be readily detected in CD11b^+^ cells, FAT10 expression in CD19^+^ is low and can barely be detected in cytotoxic T cells (7).

## Absence of FAT10 does not alter presentation of different endogenous and virus derived MHC-I peptides

**Figure 5:**
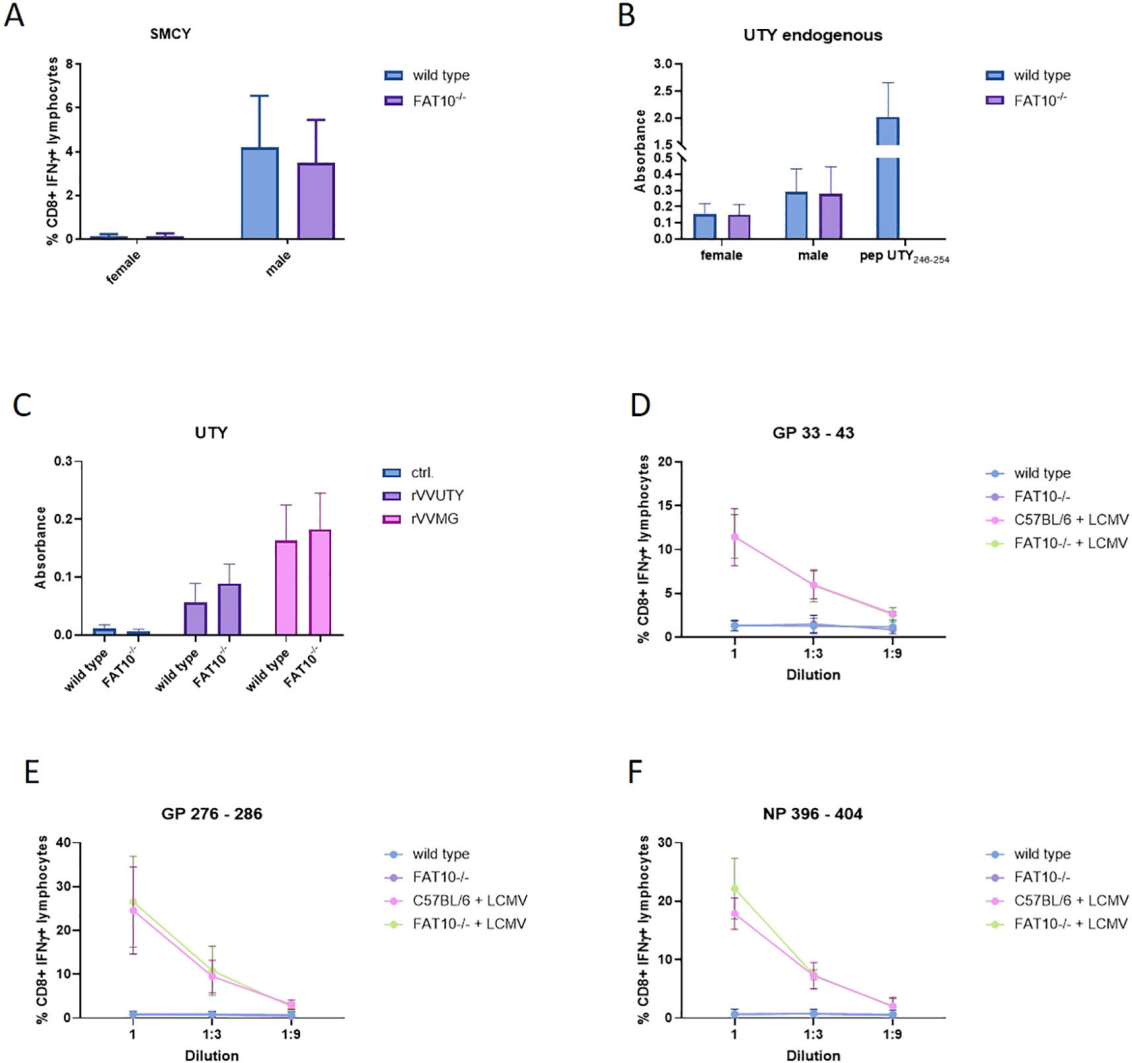
Effect of FAT10-deficiency on presentation of endogenous and viral MHC-I epitopes. **(A)** SMCY_738–746_ presentation on macrophages derived from female or male wild type mice or FAT10^-/-^mice was analyzed with SMCY_738–746_ specific CTL lines. Activation of CTL-lines was analysed by staining for CD8 and intracellular IFNγ. Shown are the percentages of IFNγ-positive cells of CD8^+^ lymphocytes as determined by flow cytometry. Female cells were used as negative control. Data are shown as mean ± SD from 3 independent experiments measured in triplicates. **(B)** The presentation of UTY_246–254_ on macrophages derived from male or female (negative control) wild type mice or FAT10-deficient mice was determined with a UTY_246–254_-specific T cell hybridoma in chromogenic lacZ assays. The y-axis shows absorbance of enzymatically converted chromogen at 570 nm in lacZ assays. The values depicted as means ± SD of 3 experiments measured in triplicate cultures. **(C)** Macrophages derived from female wild type mice or FAT10-deficient mice were infected with recombinant vaccinia viruses expressing the full length UTY-protein (rVVUTY) or UTY_246–254_ as a minigene (rVVMG). The presentation of UTY_246–254_ was determined with a UTY_246–254_-specific T cell hybridoma. Secretion of IL-2 into the supernatant was measured by ELISA. The y-axis shows absorbance of enzymatically converted substrate in ELISA. The values are depicted as mean ± SD of 4 experiments measured in triplicate cultures. **(D–F)** Comparison of the presentation of the LCMV epitopes **(D)** GP_33-41_, **(E)** GP_276-286_, and **(F)** NP_396–404_ by IFN-γ-treated and LCMV-infected MEFs derived from wild type mice or FAT10-deficient mice. Infected cells were used as stimulators for GP_33-41_-, GP_276-286_-, and NP_396-404_-specific CTL lines. Activation of CTL-lines was analyzed by staining for CD8 and intracellular IFNγ. Shown are the percentages of IFNγ-positive cells of CD8^+^ cells as determined by flow cytometry. The percentage of IFNγ^+^ of CD8^+^ cells (y-axis) is plotted versus the E:S ratio [effector (CTL lines) to stimulators (MEFs)]. Uninfected MEFs derived from wild type or FAT10^-/-^ cells were used as negative controls. Data are shown as mean ± SD from 4 independent experiments measured in duplicates.

Next, we aimed to investigate whether the ubiquitin-like modifier FAT10 influences the generation of specific peptides presented on MHC class I molecules. We assessed the antigen presentation capabilities of macrophages and mouse embryonic fibroblasts (MEFs) derived from both wild type and FAT10 knockout mice. Cells were stimulated for one day with IFNγ/TNF to induce FAT10. SMCY and UTY are proteins encoded on the Y chromosome resulting in an endogenous expression only in cells derived from male mice (27–29). First, we used specific T cells to evaluate the expression of SMCY_738-746_/D^b^. Therefore, we used *in vitro* restimulated T cells from female mice carrying a transgenic T cell receptor designed to recognize the SMCY-derived peptide SMCY_738-746_ (30). Activation of SMCY_738–746_ specific T cell lines by macrophages derived from female/male wild type mice or FAT10^-/-^ mice was assessed by intracellular IFNγ staining and flow cytometry (**Figure 5A**). Whereas female macrophages were not recognized, male macrophages derived from wild type mice or FAT10-deficient mice stimulated SMCY_738–746_ specific T cells in a similar manner. Hence, FAT10 does not affect presentation of SMCY_738-746_. Next, the influence of FAT10 on UTY_246–254_ presentation was investigated. Therefore, UTY_246-254_-specific T-cell hybridomas were used. Upon antigen specific activation, UTY T-cell hybridomas produce β-galactosidase and secrete IL-2. Male and female macrophages derived from C57BL/6 mice or FAT10-deficient mice were used to stimulate UTY T-cell hybridomas (**Figure 5B**). Female macrophages indicate background activation of UTY T cell hybridomas. Male macrophages stimulated UTY T-cell hybridomas above background levels. However, no difference between wild type and FAT10-deficient macrophages could be observed. To confirm these results, we expressed the UTY protein with the help of a recombinant vaccinia virus encoding UTY in female macrophages. A recombinant vaccinia virus encoding the UTY_246–254_ epitope as mini-gene served as control. No difference in UTY_246–254_ presentation could be observed between wild type cells and FAT10-deficient cells (**Figure 5C**). Next, we investigated the effect of FAT10 in the presentation of three virus derived MHC-I peptides. Therefore, wild type or FAT10-deficient mouse embryonic fibroblasts (MEFs) were infected with lymphocytic choriomeningitis virus (LCMV). Activation of CTL-lines specific for GP33-41/K^b^/D^b^, GP276-286/D^b^ and NP396-404/D^b^ was used to detect LCMV-specific MHC-I presentation (31, 32). Stimulation with synthetic peptides was used to determine specificity and maximal activation of CTL-lines (**Supplementary Figure 2**). No significant differences in presentation of three LCMV-derived peptides were observed between wild type and FAT10-deficient MEFs (**Figures 5D–F**). Taken together, the absence of FAT10 did not alter the MHC-I antigen presentation of different immunogenic peptides on murine cells, suggesting that FAT10 does not play a crucial role in the generation or presentation of peptides on MHC class I molecules.

## FAT10-deficiency does not alter the virus-specific cytotoxic T cell response

**Figure 6:**
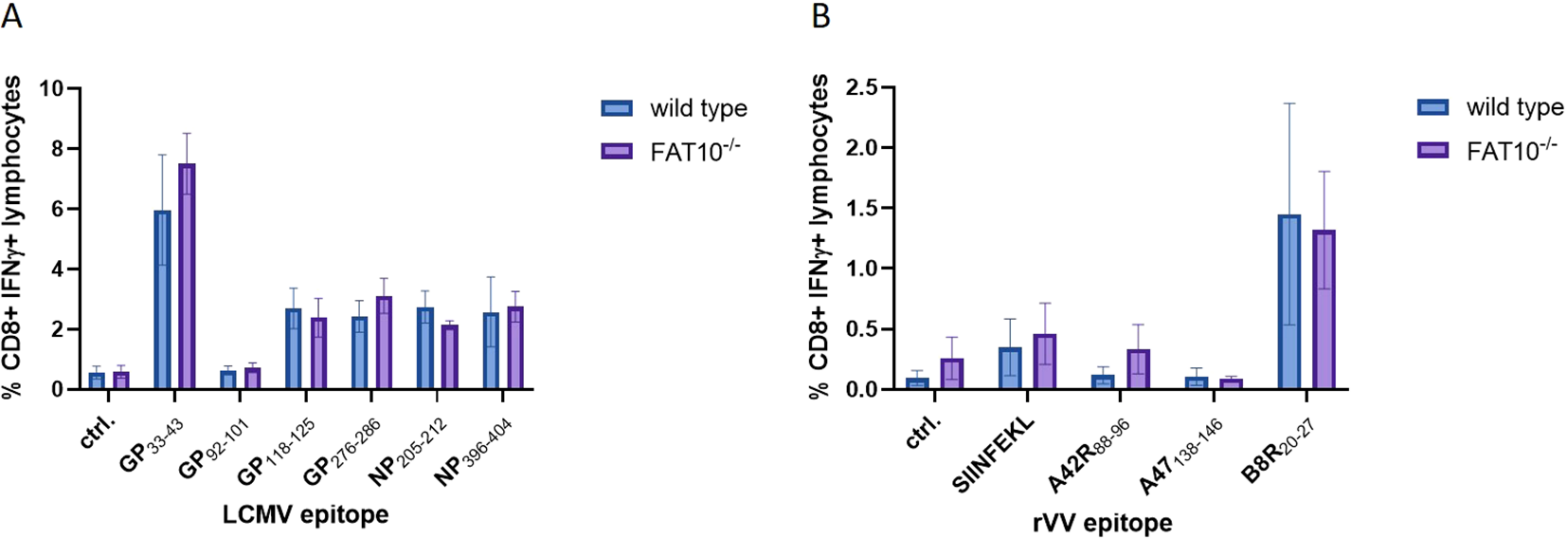
CTL response in LCMV and vaccinia virus infected FAT10-deficient mice. **(A, B)** C57BL/6 mice or FAT10 KO mice were infected with LCMV **(A)** or vaccinia virus expressing ovalbumin **(B)**. 8 days post infection, splenocytes were re-stimulated *in vitro* with immunogenic peptides of the respective virus for 5 h Activation of peptide specific T cells was determined by staining for CD8 and intracellular IFNγ and analyzed by flow cytometry. The percentages of IFNγ-positive cells of CD8^+^ cells are shown. Data are shown as mean ± SD from 3 independent experiments measured in triplicates.

Previous experiments showed that FAT10 depletion does not affect MHC-I antigen presentation in murine and human cells, indicating no crucial function in the generation of the peptide pool for MHC class I presentation. The peptide presentation on MHC-I is a crucial initial step in the induction of a cytotoxic T cell response. Hence, a putative influence of FAT10 on MHC-I antigen presentation should finally lead to an altered cytotoxic T cell response. Therefore, we used two different virus models with well-characterized MHC class I restricted epitopes. Wild type and FAT10 knock-out mice were immunized with LCMV or recombinant vaccinia virus encoding ovalbumin. On day 8 post immunization, splenocytes were isolated and re-stimulated *in vitro* with different virus specific immunogenic peptides. It has been shown that infection with viruses (LCMV, influenza virus) strongly induces the expression of FAT10 (33). The activation of T cells was determined by measuring the proportion of IFNγ-secreting CD8 positive lymphocytes by flow cytometry (**Figure 6**). The LCMV-specific cytotoxic T cell (CTL) response was dominated by GP_33-41_, whereas CTL response to GP_92–101_ was barely above background levels. For all LCMV epitopes analyzed, no significant differences in CTL activation between wild type and FAT10 knockout T cells could be observed (**Figure 6A**). Next, the CTL response in vaccinia virus infected mice was investigated (**Figure 6B**). Notably, the anti vaccinia virus CTL response was dominated by B8R_20–27_ specific CTLs, whereas A42R_88–96_ and A47_138–146_ specific CTLs were not detected. Similar to the findings with LCMV, FAT10 deficiency did not influence the CTL response to vaccinia virus. In summary, FAT10 deficiency did not show an altered CTL response specific to different LCMV and vaccinia virus epitopes. Hence, we conclude that the ubiquitin-like modifier FAT10 has no essential role in generating peptides for MHC-I antigen presentation.

## Discussion

The proteasome is crucial for the degradation of proteins and the presentation of viral epitopes on MHC class I molecules (1, 34). Ubiquitin targets proteins for proteasomal degradation. Although the requirement of protein ubiquitylation for MHC-I peptide generation is generally assumed, contradictory results have been obtained (35–43). Thus, it has been proposed that ubiquitin-dependent and -independent pathways robustly contribute to MHC class I-based immunosurveillance (35). This suggests the existence of alternative molecules targeting proteins for proteasomal degradation. Similar to ubiquitin, posttranslational modification with the ubiquitin-like modifier FAT10 targets its substrate for proteasomal degradation (3, 4, 22, 23, 44). Hence, it seems reasonable that in addition to ubiquitin, FAT10 can contribute to the generation of MHC-I ligands. Moreover, FAT10 is expressed in organs of the immune system and is upregulated during an inflammatory response (5, 7). Therefore, FAT10 might act as an alternative proteasome-targeting signal for immune cells, thereby increasing the MHC-I peptide pool during infection. Indeed, it could be shown that N-terminal fusion of FAT10 to different viral proteins enhances the presentation of peptides derived from these proteins on MHC class I *in vitro* (23–25). Using different human cell lines, our data shows that the number of MHC class I molecules on the cell surface remains unchanged in the absence of FAT10 (**Figures 1**, **2**). Simulating inflammation by adding TNF and IFNγ to the cells enhanced the overall level of MHC molecules, especially pronounced in A549 cells. However, no influence of FAT10 could be observed (**Figure 2E**). Similarly, the *de novo* formation of peptide:MHC I complexes on the cell surface was not affected by the absence of FAT10 (**Figure 3**). Furthermore, the levels of the total murine MHC class I molecules H-2D^b^ and H-2K^b^ were unaltered across different immune cell populations derived from FAT10^-/-^ mice (**Figure 4**). Similar to bulk MHC-I surface expression, the MHC class I presentation of different endogenous and virus-derived immunogenic peptides remained unaffected in the absence of FAT10 in mice (**Figure 5**). It should be emphasized that the findings of this study are based on a defined set of model antigens. These antigens are well-characterized and highly immunogenic, which allowed us to specifically assess the impact of FAT10 on antigen presentation. When considered alongside data from the analysis of bulk MHC molecules, our findings suggest that FAT10 does not broadly influence antigen presentation, extending beyond the specific model antigens used in this study. Alteration in MHC-I antigen presentation influences the induction of a CTL response (34). However, no influence of FAT10 in the CTL response to LCMV or vaccinia virus could be observed in infected mice. It has been shown that N-terminal fusion of either ubiquitin or FAT10 to the long-lived nucleoprotein of LCMV accelerates its degradation by the proteasome (23). Thereby, FAT10 increases the generation of epitopes eligible for loading onto MHC molecules. Held et al. could show that N-terminal conjugation of ISG15 to the nucleoprotein of LCMV leads to an enhanced antigen-presentation of LCMV-derived epitopes (24). The ISG15 fusion protein acts as a degron thereby enhancing the generation of peptides that can be presented on MHC class I molecules. Similar to this, FAT10-conjugated LCMV-nucleoprotein undergoes accelerated degradation via the proteasome (24). Furthermore, the N-terminal fusion of the human CMV-derived pp65 antigen to FAT10 facilitated direct MHC-I-restricted presentation and DC-mediated cross-presentation of the HLA-A2–restricted pp65_495–503_ epitope (22). Notably, in all these studies showing an effect of FAT10 on MHC-I presentation, an artificial fusion protein was introduced to be processed by the proteasome resulting in an increased peptide pool for MHC class I presentation. However, in our study we investigated the role of FAT10 under physiological conditions. N-terminal conjugation of ubiquitin, FAT10 or ULMs in common is a frequently used method to study the role of these modifiers in biochemical and cellular processes. Unlike ubiquitin, a single molecule of FAT10 is sufficient for mediating proteasomal degradation (3). However, N-terminal FAT10 conjugation does probably not accurately reflect the physiological conjugation process occurring in the cell. FAT10 is conjugated via an isopeptide linkage to an ϵ-amino group of a lysine residue within the substrate. Covalent FAT10-modified proteins are targeted together with FAT10 for degradation by the 26S proteasome. Moreover, only a small percentage of proteins is FAT10ylated and the abundance of FAT10 in cells is relatively small in comparison to ubiquitin. Consequently, ubiquitin predominates, leading to limited FAT10 conjugation. Indeed, only 5-10% of the entire protein pool is FAT10ylated (45, 46). Thus, FAT10ylation happens rather rarely compared to ubiquitination. It is also noteworthy that FAT10 can be conjugated non-covalently suggesting additional functions besides targeting proteins to the 26S proteasome (47, 48). Although N-terminal conjugation of FAT10 leads to an enhanced presentation of the nucleoprotein-derived MHC class I epitopes NP_396–404_ or NP_118–126_ (23, 24), no influence of FAT10 could be observed in our study not using fusion proteins (**Figures 5E–F**). Accordingly, the absence of FAT10 did not affect the cytotoxic T cell (CTL) response to viral epitopes in mice immunized with either LCMV or recombinant vaccinia virus (**Figure 6**). Interestingly, vaccination with N-terminal FAT10 does not improve the immunogenicity of DNA vaccines and recombinant VVs (23).

Although N-terminal FAT10 conjugation increases degradation and antigen presentation *in vitro*, the absence of FAT10 does not impact direct MHC-I antigen presentation and CTL responses *in vivo* in our study. Hence, our data suggest that the contribution of FAT10 to MHC-I antigen presentation has previously been overestimated (17, 23, 47, 49). Mechanistically, it is unlikely that FAT10 plays a role in MHC-I antigen processing in viral infection. In contrast to ubiquitin, FAT10 expression is specifically induced by the pro-inflammatory cytokines TNF and IFNγ, which are predominantly secreted by CTLs during viral infection. Considering the temporal dynamics of infection, FAT10 upregulation occurs at a later stage when antigen presentation by dendritic cells and priming of T cells have already occurred, arguing against a critical role for FAT10 in this process. Additionally, considering a substantial amount of substrates that interact non-covalently with FAT10, indicates that its functions extending targeted degradation may be of greater relevance (45, 46, 48). The ULM can modify proteins in ways that influence their function, localization, and interaction with other cellular components (47). An example of non-covalent interaction is observed with the spindle checkpoint protein MAD2, which becomes sequestered by FAT10, leading to chromosomal instability (50). Furthermore, FAT10 can interact non-covalently with proteins to inhibit their degradation via ubiquitin, as for the transcription factor β-catenin (51). This particular interaction results in an aberrant accumulation of active β-catenin, which promotes cellular proliferation (51). Thus, FAT10 influences cell division, which in turn may contribute to tumor progression. Additionally, FAT10 was shown to modulate immune responses through non-covalent interactions. Binding of FAT10 to RIG-I impairs RIG-I-mediated antiviral signaling (52, 53). Moreover, the interaction of FAT10 with OTUB1 stabilizes the deubiquitylating enzyme, leading to the down-regulation of type-I interferon production, a key component of the antiviral response (46). Consequently, in addition to regulating protein turnover through degradation by the 26S proteasome, FAT10 plays a more crucial role in other processes such as interferon regulation (33, 54, 55) or tumor development (13, 56).

## Materials and methods

### Mice

C57BL/6 mice (H-2b) were originally purchased from Charles River, Germany. FAT10-deficient (FAT10^-/-^) mice (18) were kindly provided by A. Canaan and S.M. Weissman (Yale University School of Medicine, New Haven, USA) and backcrossed onto C57BL/6 background for at least 10 generations. 8–10 week old mice were used for all experiments. HY TCR-tg mice (57) were obtained from A. Tafuri (Deutsches Krebsforschungszentrum, Heidelberg, Germany). Mice were infected intravenously (i.v.) with 200 pfu LCMV-WE. For infection with recombinant vaccinia virus, mice were infected intraperitoneally (i.p.) with 200 pfu of the VV-OVA (recombinant vaccinia viruses (VV) encoding the ovalbumin (provided by Y. Yewdell, National Institute of Allergy and Infectious Diseases, Bethesda, MD, USA)) or VV-UTY (recombinant vaccinia viruses (VV) encoding the Uty gene (rVV-UTY) (provided by V. Cerundulo, University of Oxford)). Animal experiments were approved by the Review Board of Governmental Presidium Freiburg of the State of Baden-Württemberg, Germany (G-12/114, I-13/01, G-15/112, G-18/72, I-18/03, I-21/001). Animals were sacrificed by cervical dislocation or CO_2_ euthanasia (30-40% flow rate of chamber volume per minute) using the GasDocUnit^®^ (medres medical research, Germany). The GasDocUnit^®^ is in accordance with the American Veterinary Medical Association (AVMA) guidelines for the euthanasia of animals, the German animal law and the European Union guidelines 2010/63/EU.

### Cell culture

RPMI, IMDM or DMEM media were supplemented with 10% (v/v) FBS, 100 U/mL penicillin and 100 µg/mL streptomycin. Murine embryonic fibroblasts (MEFs) derived from C57BL/6 mice or FAT10-deficient mice were generated as previously described (58). HEK293, A549, HepG2 and their respective FAT10 knock-out cell lines [HEK293 FAT10KO (59), A549 FAT10KO (60), HepG2 FAT10KO (59)] were cultured in DMEM. HCT116 and its FAT10 knock-out cell line (59) was cultured in RPMI. UBA6 and USE1 knock-out cell lines were generated as previously described (45, 61) and cultured in IMDM. Cells were grown at 37°C and 5% CO_2_. All wild type cell lines (HEK293, A549, HepG2, HCT116) were originally obtained from ATCC. For cytokine treatment, cells were stimulated with 200 U/ml IFNγ (peprotech) and 400 U/ml TNF (peprotech). After incubation at 37°C and 5% CO_2_, cells were used for experiments. Peritoneal macrophages were generated by i.p. injection of 0.5 ml 3% thioglycolate broth. After 3–4 days, cells were washed out of the abdominal cavity by peritoneal lavage using PBS.

### Acid wash

Cells were treated with citric acid buffer (0.131 M citric acid, 0.066 M NaH_2_PO_4_, pH 3) for 1 minute to remove MHC-I surface molecules. Cells were washed twice with ice cold PBS and medium. Cells were further incubated at 37°C and 5% CO_2_ for the time period indicated in the experiment. MHC class I expression was analyzed via flow cytometry. Therefore, cells were stained with anti HLA-A,B,C (W6/32, BioLegend). Measurements were performed using a FACSVerse flow cytometer (BD Biosciences) and analyzed using FlowJo software (BD Biosciences).

### MHC-I surface of splenocytes

Splenocytes derived from C57BL/6 wild type mice or FAT10-deficient mice were stained for CD8 (53-6.7, BioLegend), CD19 (1D3/CD19, BioLegend), or CD11b (M1/70, BioLegend) and H-2D^b^ (KH95, BioLegend) or H-2K^b^ (AF6-88.5, BioLegend) for 20 min at 4°C. Measurements were performed using a BD Accuri C& (BD Biosciences).

Immunoprecipitation, SDS-PAGE and western blotting

Lysis, SDS-PAGE and western blotting was performed as previously described (62). Shortly, cells were lysed in 100 µL lysis buffer (1% Triton X-100, 10 mM Tris pH 6,8, 150 mM NaCl) and incubated on ice for 30 minutes. Lysates were centrifuged for 20 minutes at full-speed at 4°C in a tabletop centrifuge. Cleared lysates were incubated with 1x Laemmli sample buffer (50 mM Tris-HCl pH 6.8, 2% SDS, 10% glycerol, 20 mM dithiothreitol, 0.02% bromophenol blue) for 5 min at 95°C. For immunoprecipitation, lysates were incubated with 30 µl pre-equilibrated protein A-sepharose beads (HepG2, Hek293) or pre-equilibrated protein G-sepharose beads (A549, HCT116) and 5 µg of monoclonal FAT10-reactive antibody 4F1 (10) were added to the cell lysate. After incubation overnight at 4°C, samples were washed twice with 1 mL NET-TN (50 mM Tris-HCl pH 8.0, 650 mM NaCl, 5 mM EDTA, 0.5% (v/v) Triton X-100) and twice with NET-T (50 mM Tris-HCl pH 8.0, 150 mM NaCl, 5 mM EDTA, 0.5% (v/v) Triton X-100) buffer. Proteins were separated according to their mass by SDS-PAGE and subsequently transferred onto a nitrocellulose membrane (Whatman). Membranes were blocked for 1 h at room temperature using Intercept Blocking Buffer (LI-COR) and incubated with primary antibodies at 4°C over night. Next, membranes were washed and incubated with appropriate secondary antibodies. Secondary antibodies used were: IRDye 680RD goat anti-mouse IgG and IRDye 800CW goat anti-rabbit IgG (both LI-COR). Afterwards, proteins were visualized using Odyssey Fc Imaging System (LI-COR) and quantified using ImageStudio (Ver. 5.2, LI-COR).

### LacZ assay

The LacZ assay was performed as previously described (63). Briefly, 10^5^ cells of the UTY_246–254_-specific T-cell hybridoma (kindly contributed by Nilabh Shastri, University of California, Berkeley, CA) were co-cultured with 1.5 × 10^6^ stimulator cells in 96-well plates overnight. The lacZ-based color reaction was performed and measured as described in (64).

### Generation of CD8-specific T cells

CD8-specific T cells were generated as described previously (65). Briefly, naive C57BL/6 mice at 6 to 8 weeks of age were intravenously (i.v.) infected with 200 PFU LCMV-WE (kind gift of Maries van den Broek, University of Zurich, Switzerland). Four weeks after infection, mice were sacrificed and spleens were homogenized. Splenocytes were further cultured in 6-well plates and directly pulsed with 10^–6^ m of the respective peptide supplemented with 40 U/mL IL-2. The medium was renewed after two days. On day 5, Ficoll gradient centrifugation was performed to remove dead cells. Remaining splenocytes were further cultured in the presence of 40 U/mL IL-2. LCMV-specific CD8 T-cells were used between days 7 and 9 of culture.

### *In vitro* antigen presentation assays and intracellular cytokine staining (ICS).

To evaluate the direct antigen presentation of FAT10 deficient cells, the activation of CD8^+^ T-cells was measured in an *in vitro* assay. For the presentation of the SMCY peptide, macrophages isolated from male mice were used. LCMV-derived epitopes were presented by mouse embryonic fibroblasts (MEFs). Therefore, MEFs were stimulated with 100 U/ml IFNγ and 200 U/ml TNF for 48 h before infection with LCMV-WE (MOI = 10) for 3 h. Afterward, cells were seeded in a 96-well plate in serial 3-fold dilutions starting with 4 x 10^5^ cells. Virus-specific CD8^+^ T-cells (2 × 10^5^) and brefeldin A at a final concentration of 10 μg/mL were added. Samples were incubated for 5 h at 37°C. To analyze the CTL-response, splenocytes were incubated in the presence or absence of 10^–5^ M synthetic peptides and brefeldin A (10 μg/mL) for 5 h. Cells were fixed using 4% paraformaldehyde and stained using the following antibodies: anti-CD8a (53-6.7, eBioscience), anti-IFNγ (XMG1.29, eBioscience). Measurements were performed using a FACSVerse flow cytometer (BD Biosciences) and analyzed using FlowJo software (BD Biosciences). For analysis FlowJo software (BD Biosciences) was used.

### Statistic

For statistical analyses, groups from three or four independent experiments were pooled and analyzed for significant differences as indicated in the figure legends. Statistical significance was determined using GraphPad Prism software (version 9.5.1. GraphPad, San Diego, CA). Error bars represent mean ± SD unless otherwise stated.

## Data Availability

The raw data supporting the conclusions of this article will be made available by the authors, without undue reservation.
