# Supplementary material for: The ubiquitin-like modifier FAT10 is not essential for MHC-I antigen presentation
Source: Front Immunol. 2025 Aug 1;16:1636951. doi: 10.3389/fimmu.2025.1636951 (PMC12353742; doi:10.3389/fimmu.2025.1636951)
Supplement: Supplementary file 1 [file DataSheet1.docx]

**Supplementary Information**

**The ubiquitin-like modifier FAT10 is not essential for MHC-I antigen presentation**

Natalie Pach^1,2,#^, Sarah Ochs^2,#^, Jinjing Cao^2.#^, Julia Ottlinger^1^, Annette Aichem^1^, Michael Basler^1,2,*^

^1^ Institute of Cell Biology and Immunology Thurgau (BITG) at the University of Konstanz, CH-8280 Kreuzlingen, Switzerland

^2^ Division of Immunology, Department of Biology, University of Konstanz, D-78457 Konstanz, Germany

^#^ contributed equally

* Corresponding author: Michael Basler


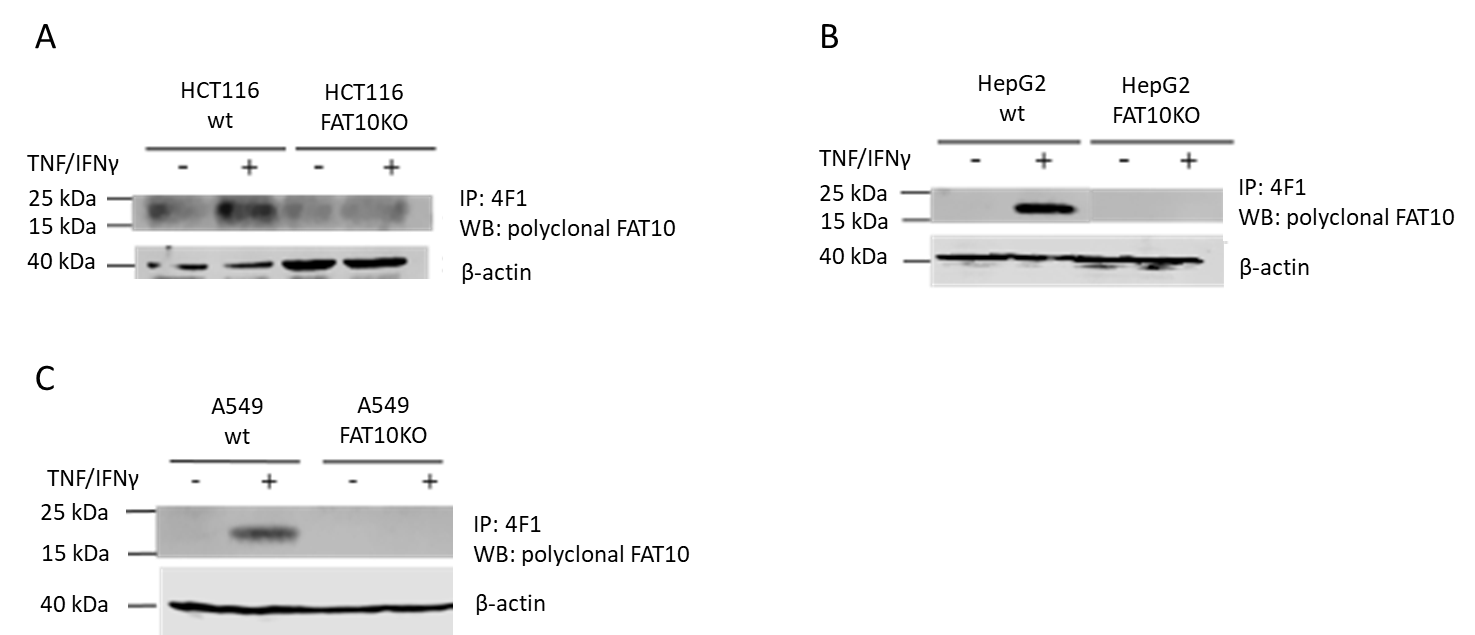


**Supplementary Figure 1** Western blot analysis of different FAT10 KO cell lines. **(A, B, C)** FAT10 was immunoprecipitated with the 4F1 antibody and analyzed by western blot using polyclonal antibodies recognizing human FAT10. Analysis was performed in wild type (wt) or FAT10-deficient (FAT10KO) (A) colon carcinoma cell line HCT116, (B) hepatocellular carcinoma cell line HepG2, and (C) lung cancer cell line A549.Where indicated, cells were stimulated with TNF/IFNγ. β-actin was used as loading control.

**Supplementary Figure 2** Activation of LCMV-specific CTL lines. Specific CTL lines were activated in the presence of the respective immunogenic LCMV-derived peptide. Activation of CTL lines was analyzed by staining for surface CD8 and intracellular IFNγ. Percentage of IFNγ positive cells of CD8 positive lymphocytes was determined by flow cytometry. Data are shown as mean ± SD from 5 independent experiments measured in duplicates.
